# Cyclic Peptide-Gadolinium Nanoparticles for Enhanced Intracellular Delivery

**DOI:** 10.3390/pharmaceutics12090792

**Published:** 2020-08-21

**Authors:** Amir Nasrolahi Shirazi, Shang Eun Park, Shirin Rad, Luiza Baloyan, Dindyal Mandal, Muhammad Imran Sajid, Ryley Hall, Sandeep Lohan, Khalid Zoghebi, Keykavous Parang, Rakesh Kumar Tiwari

**Affiliations:** 1 Department of Pharmaceutical Sciences, College of Pharmacy, Marshall B. Ketchum University, Fullerton, CA 92831, USA; ShirinRad.PH23@ketchum.edu (S.R.); LuizaBaloyan.PH23@ketchum.edu (L.B.); 2 Center for Targeted Drug Delivery, Department of Biomedical and Pharmaceutical Sciences, Chapman University School of Pharmacy, Harry and Diane Rinker Health Science Campus, Irvine, CA 92618, USA; park327@mail.chapman.edu (S.E.P.); sajid@chapman.edu (M.I.S.); hall222@mail.chapman.edu (R.H.); lohan@chapman.edu (S.L.); zoghe101@mail.chapman.edu (K.Z.); parang@chapman.edu (K.P.); 3 School of Biotechnology, KIIT Deemed to be University, Bhubaneswar 751024, India; dmandal@kiitbiotech.ac.in; 4 Faculty of Pharmacy, University of Central Punjab, Lahore 54000, Pakistan

**Keywords:** drug delivery systems, cyclic peptides, gadolinium nanoparticles, intracellular transportation, nanocarriers

## Abstract

A cyclic peptide containing one cysteine and five alternating tryptophan and arginine amino acids [(WR)_5_C] was synthesized using Fmoc/tBu solid-phase methodology. The ability of the synthesized cyclic peptide to produce gadolinium nanoparticles through an in situ one-pot mixing of an aqueous solution of GdCl_3_ with [(WR)_5_C] peptide solution was evaluated. Transmission electron microscopy showed the formed peptide-Gd nanoparticles in star-shape morphology with a size of ~250 nm. Flow cytometry investigation showed that the cellular uptake of a cell-impermeable fluorescence-labeled phosphopeptide (F′-GpYEEI, where F′ = fluorescein) was approximately six times higher in the presence of [(WR)_5_C]-Gd nanoparticles than those of F′-GpYEEI alone in human leukemia adenocarcinoma (CCRF-CEM) cells after 2 h incubation. The antiproliferative activities of cisplatin and carboplatin (5 µM) were increased in the presence of [(WR)_5_C]-GdNPs (50 μM) by 41% and 18%, respectively, after 72-h incubation in CCRF-CEM cells. The intracellular release of epirubicin, an anticancer drug, from the complex showed that 15% and 60% of the drug was released intracellularly within 12 and 48 h, respectively. This report provides insight about using a non-toxic MRI agent, gadolinium nanoparticles, for the delivery of various types of molecular cargos.

## 1. Introduction

Metal-containing materials have been used extensively in designing various biomedical projects such as imaging cargos, detection markers and therapeutics [1,2]. Optimized functionality of metal-containing materials could be obtained by utilizing various elements such as size, active surface, morphology and stability properties. Therefore, researchers have made their best effort to tailor the orientation of functional groups in drug delivery systems to achieve their optimized function [3,4].

A member of the lanthanide family, gadolinium (Gd) has exhibited extensive potency for clinical applications due to its unparalleled chemical properties [5]. Gadolinium offers a paramagnetic property due to the presence of seven unpaired electrons in the 4f shell, which makes it a potential agent for magnetic resonance imaging (MRI) [6]. It enhances MR images by reducing *T*_1_ relaxation constant of the tissues in which accumulated. Gd has been shown to be able to chelate to various types of ligands and form stable complexes. Gd-DTPA (Magnevist) and Gd-DOTA (Dotarem) are examples of commercially available products that are being used as *T*_1_ contrast agents. Although Gd containing complexes have been used widely, they suffer from leaching issues where non-metabolizable Gd^3+^ ions could potentially induce toxicity through accumulation in the body. Excessive Gd^3+^ toxicity could lead to renal toxicity and inhibition of calcium channels in patients with insufficient renal function [7]. Therefore, discovering novel Gd-containing complexes with insignificant toxicity could be of potential interest for researchers.

To date, gadolinium-based nanoparticles showed promising potential, and they have been approved by the FDA for MRI imaging in cancer patients [8]. To improve Gd^3+^ leakage, carriers with a higher degree of stability have been introduced [9]. In addition, liposome-based carriers containing Gd have been found to have potential for improved tumor diagnosis [10].

In addition to the use of gadolinium for clinical diagnosis procedures, recently, complexes containing contrast agents and drugs have been designed as potential platforms for drug delivery and imaging applications. For instance, gadolinium-based cancer therapeutic liposomes have been utilized to improve diagnosis and chemotherapy outcomes [11,12]. Dual functional liposomes were prepared to encapsulate both doxorubicin and Gd via drug-metal complexation. The complex was found to be potent in terms of a high accumulation of Gd nanoparticles in target tissues and a higher degree of doxorubicin internalization. Similar studies were performed by fabricating Gd-doped layered double hydroxide/Au nanocomposite. The synthesized nanoparticles showed high doxorubicin loading capacity with a pH-responsiveness release profile. They were found to be able to release doxorubicin in the cytoplasm [13]. In addition to the release profile, the loading of the molecular cargo into the Gd-containing delivery system has been a challenging task. One of the most efficient methods is taking advantage of π–π stacking interaction between the cargos and delivery systems [14]. This method can be properly used for hydrophobic cargos containing benzene rings or similar scenarios for the delivery systems. Generating a hydrophobic pocket using certain amino acids is one of the methods to entrap the cargos. Additionally, a stimuli-responsive polymeric prodrug-based nanotheranostic system with imaging agent (cyanine 5.5 and gadolinium-chelate) and a therapeutic agent paclitaxel (PTX) were fabricated. The imaging-guided chemotherapy investigations to the 4T1 tumor in the mice model showed an outstanding anti-tumor effect [15].

A peptide-based drug delivery system is one of the extensively used tools to overcome major obstacles in permeation into cells. In recent years, synthetically engineered cyclic peptides have been utilized as drug delivery systems for transporting various types of molecular cargos such as anticancer drugs and siRNAs [16]. The cyclic nature of peptides has been discovered to be critically important to enhance their intracellular transporting efficiency [17,18,19]. We introduced a class of homochiral L-cyclic peptides with enhanced potency in nuclear targeting delivery of anti-HIV drugs and biomolecules [20]. Unlike linear peptides, cyclic counterparts were found to be offering a preferred orientation of guanidine groups and positive charge distribution on the surface of the peptide leading to a higher level of cell permeability [20,21,22]. Additionally, the rigidity in the structure of peptides that are obtained through cyclization makes them more stable in the presence of peptidases [23,24,25,26]. 

Although the synthesized cyclic peptides were found to be transporting drugs into cells efficiently, their ability was improved exponentially when mixed with metal nanoparticles such as gold and selenium [27,28,29]. The presence of arginine and tryptophan amino acids in their structures were highly important to interact with positively charged metal ions and the cell membrane elements at the same time. The presence of tryptophan and arginine amino acids in the structures of peptides induced binding to metal nanoparticles through electrostatic and hydrophobic forces [30]. Therefore, various types of metals, in combination with peptides that can be used in drug delivery systems, have become subjects of major interest. 

In this work, we designed a drug delivery system containing both cyclic cell-penetrating peptide and gadolinium (Gd) nanoparticles (CP-GdNPs). Gadolinium has already proved to be a convenient choice for imaging due to its chemical and physical properties and high penetration. Here, we took advantage of the unique nature of gadolinium and combined it with the bio-friendly nature of peptides to introduce a new class of gadolinium-peptide-based drug delivery systems for carrying various types of molecules. The ability of CP-GdNPs was evaluated for the delivery of a relatively large biomolecule (a negatively charged phosphopeptide) in cell-based assays. 

## 2. Materials and Methods 

All amino acids and resins were purchased from AAPPTec, LLC (Louisville, KY, USA). All organic solvents and reagents were purchased from Millipore Sigma Corporation (St. Louis, MO, USA) and Fisher Scientific (Pittsburgh, PA, USA). Molecular weights of intermediate and final products were confirmed by high-resolution matrix-assisted laser desorption ionization time-of-flight (MALDI-TOF) mass spectrometer from Bruker Inc. (GT 0264, Billerica, MA, USA). Intermediate and final compounds were purified by reversed-phase high-performance liquid chromatography (RP-HPLC) from Shimadzu (Prominence, Columbia, MD, USA) using a gradient system of acetonitrile and water with 0.1% trifluoroacetic acid (TFA) using a reverse-phase column (XBridge BEH C18 OBD Prep Column), from Waters Corporation (Milford, MA, USA). 

### 2.1. Synthesis of Cyclic Peptide 1 [(WR)_5_C]

The cyclic peptide containing eleven amino acids [CWRWRWRWRWR] was synthesized by Fmoc/tBu solid-phase peptide synthesis. Arg(Pbf)-OH preloaded on 2-chlorotrityl resin (0.3 mmol, 524 mg, 0.572 mmol/g) was swollen in *N,N*-dimethylformamide (DMF) using agitation with purging nitrogen. During the synthesis of the peptide, the Fmoc group was deprotected using 20% piperidine in DMF (*v*/*v*) under nitrogen (20 min × 2). Coupling of each amino acid were performed using fmoc and side chain-protected amino acids (0.9 mmol) using 2-(1H-benzotriazol-1-yl)-1,1,3,3-tetramethyluronium hexafluorophosphate (HBTU, 341 mg, 0.9 mmol, 3 equiv.) and diisopropylethylamine (DIPEA, 313 μL, 1.8 mmol) in DMF (15 mL) as coupling and activating reagents, respectively, for 90 min. After every step (deprotection or coupling), the peptidyl resin was washed using DMF (20 mL × 3) and excess solvent from resin were drained. The cycle of deprotection and coupling were followed based on the sequence of peptide **1**, e.g., in the order: Fmoc-Trp(Boc)-OH (474 mg, 0.9 mmol), Fmoc-Arg(Pbf)-OH (584 mg, 0.9 mmol), Fmoc-Trp(Boc)-OH (474 mg, 0.9 mmol), Fmoc-Arg(Pbf)-OH (584 mg, 0.9 mmol), Fmoc-Trp(Boc)-OH (474 mg, 0.9 mmol), Fmoc-Arg(Pbf)-OH (584 mg, 0.9 mmol), Fmoc-Trp(Boc)-OH (474 mg, 0.9 mmol), Fmoc-Arg(Pbf)-OH (584 mg, 0.9 mmol), Fmoc-Trp(Boc)-OH (474 mg, 0.9 mmol) and Fmoc-Cys(Trt)-OH (527 mg, 0.9 mmol). The *N*-terminal Fmoc group was deprotected using 20% piperidine in DMF (20 min × 2). The peptidyl resin was used for cyclization by agitation with freshly prepared cleavage cocktail, dichloromethane: 2,2,2-trifluoroethanol:acetic acid (DCM:TFE:CH_3_COOH; 7:2:1, *v*/*v*/*v*, 15 mL) for 1.5 h. The filtrate was collected and evaporated completely and redissolved using 50 mL of DCM and evaporated. Cyclization was performed using anhydrous DMF and DCM (50:50, *v*/*v,* 100 mL) using *N,N’*-diisopropylcarbodiimide (DIC, 71 µL, 0.45 mmol) and 1-hydroxy-7-azabenzotriazole (HOAt, 61 mg, 0.45 mmol) for 72 h to afford cyclic peptide **1** after confirmation of mass of cyclized peptide with MALDI-TOF. The reaction mixture was evaporated under reduced pressure and the cyclic peptide was fully deprotected after stirring with freshly prepared cleavage cocktail, trifluoroacetic acid:triisopropylsilane:water (TFA:TIS:H_2_O; 90: 5:5, *v*/*v*/*v*, 13.5 mL: 750 µL: 750 µL) for 4 h. The crude cyclic peptide was precipitated by the addition of cold diethyl ether (30 mL × 3) and purified by using RP-HPLC. 

MALDI-TOF (*m*/*z*) for cyclic peptide **1** [(WR)_5_C], C_88_H_116_N_31_O_11_S: calcd, 1814.9186; found 1814.7237 [M + H]^+^.

### 2.2. In Situ Preparation of Cyclic Peptide Gd Nanoparticles [(WR)_5_C]-GdNPs

The formation reaction of gadolinium-peptide nanoparticles was carried out in aqueous media. One milliliter of concentrated solution of peptide [(WR)_5_C] (1 mM) was mixed with 1 mL aqueous solution of GdCl_3_ (1 mM) at room temperature using a vortex mixer for 2 min. The mixing was performed in a glass vial and monitored. The color or temperature of the solution did not change upon mixing.

### 2.3. Transmission Electron Microscopy (TEM) 

Samples for TEM imaging were prepared based on our previously reported method [18]. The TEM [(WR)_5_C]-GdNPs samples were prepared by placing a drop of original solution (5 µM) on an ultrathin carbon type-A 400-mesh copper grid. They were allowed to dry in the presence of air. The imaging process was conducted by EAG Laboratories (Sunnyvale, CA, USA) using FEI Techni TF-20 operated at 200 kV in brightfield TEM mode. 

### 2.4. Zeta Potential Measurement 

Zeta Potential technique was used to measure the surface charge of the nanoparticles before and after loading with the cargo phosphopeptide (GpYEEI). Malvern Nano ZS Zetasizer (Westborough, MA, USA) was used for the determination of zeta potential. The zeta potential of the complexes was determined at 40 V using disposable folded capillary cells (DTS1070). The machine was calibrated using zeta potential transfer standard DTS1235. Different concentrations of the phosphopeptide, [(WR)_5_C] and [(WR)_5_C]-GdNPs were used for the determination of individual zeta potentials using Smoluchowski approximation. All results were checked for the quality standard of the instrument. 

### 2.5. Cell Culture

Two different cell lines, human leukemia T cells (CCRF-CEM, ATCC No. CCL-119) and breast adenocarcinoma (MCF-7, ATCC No, HTB-22), were purchased from American Type Culture Collection (ATCC) (Manassas, VA, USA). Cell culture supplies were purchased from Fisher Scientific (Pittsburgh, PA, USA). Cells were proliferated on 75 cm^2^ cell culture flasks with RPMI-16 medium (for CCRF-CEM cells) and EMEM medium (for MCF-7 cells), supplemented with 10% fetal bovine serum (FBS) and 1% penicillin-streptomycin solution (10,000 units of penicillin and 10 mg of streptomycin in 0.9% NaCl) in a humidified atmosphere of 5% CO_2_ and 95% air at 37 °C. 

### 2.6. Cytotoxicity Assay of [(WR)_5_C]-GdNPs 

CCRF-CEM (50,000) cells were seeded in 96-well plates (0.1 mL/well) 24 h prior to the experiment. The old medium (EMEM containing FBS (10%)) was changed (not in case of CCRF-CEM) by treatments including [(WR)_5_C], [(WR)_5_C]-GdNPs and GdNPs (12.5–200 μM) in serum containing medium. The cells were incubated for 24 h at 37 °C in a humidified atmosphere of 5% CO_2_. The viability of cells was calculated using a SpectraMax M5 microplate spectrophotometer (Molecular Devices, San Jose, CA, USA), with the absorbance intensity at 490 nm according to our previously reported method [20].

### 2.7. Time-Dependent Antiproliferative Activity Assay

Antiproliferative activities of several anticancer drugs, including epirubicin, cisplatin, gemcitabine, etoposide, carboplatin and camptothecin, were evaluated in the presence and absence of [(WR)_5_C]-GdNPs in CCRF-CEM cells. Different time periods, including 24, 48 and 72 h, were selected. The CellTiter 96 aqueous one solution cell proliferation assay kit (Promega, Madison, WI, USA) was used for the assay. The CCRF-CEM cells (5 × 10^4^) were seeded in 100 µL of the media. The cells then placed in each well of the 96-well culture plate. The cells were incubated with drugs alone and [(WR)_5_C]-GdNPs-loaded drugs at a similar condition in triplicates. To confirm the results, DMSO (1% in water) was used as a control. To entrap the drug, the physical mixing method was used. A calculated volume of the drug solution was mixed with the desired volume of an aqueous solution of [(WR)_5_C]-GdNPs physically to obtain a final concentration of the drug (5 μM) and the [(WR)_5_C]-GdNPs (50 μM). The mixture was vortexed until the solution became homogeneous and clear. The mixture was incubated for 30 min at room temperature before adding to the cells to obtain optimized performance. The incubation of cells with their treatments was carried out at 37 °C for 24–72 h. The cell viability assay was performed by adding 20 μL of CellTiter 96 aqueous solution. The cells were incubated for another hour with the MTS reagent. The formazan absorbance was measured at 490 nm by a microplate reader. The percentage of cell survival was calculated as previously reported [20].

### 2.8. Flow Cytometry Studies

Human leukemia cells (CCRF-CEM, 1 × 10^7^) were plated in 6-well plates. Opti-MEM was selected as a medium for cells and F′-GpYEEI (5 µM) was added to all the wells. Then, a solution of [(WR)_5_C]–GdNPs (50 μM) in opti-MEM was added and cells were incubated for 2 h at 37 °C. A negative control containing F′-GpYEEI (5 µM) alone was also incubated with cells in the same plate. After 2 h, the cell treatments were removed, and cells were digested with 0.25% trypsin/EDTA (0.53 mM) for 5 min to detach any membrane-bound F′-GpYEEI and artificial surface binding. After that, PBS was used to wash the cells twice. The analysis of uptake was processed based on formerly reported techniques and settings [20].

### 2.9. Confocal Microscopy on Live Cells

The MCF-7 cells were seeded in a glass bottom petri-dish (60 mm × 15 mm) of 5000 cells/mL of opti-MEM for 24 h prior to the imaging. F′-GpYEEI was mixed and incubated with [(WR)_5_C]-GdNPs (50 µM) for 30 min at room temperature. Then, the cells were treated with a mixture of F′-GpYEEI (5 µM) and [(WR)_5_C]-GdNPs (50 µM) for 2 h at 37 °C. A solution of F′-GpYEEI (5 µM) alone was used as control. After 3 h, the medium containing the treatments was removed. The cells were washed with PBS two times. Images were acquired by the Nikon A1R confocal microscope system that provides high-resolution imaging of up to 4096 × 4096 pixels magnification for brightfield and FITC channels. Images were merged and processed using Image J software (Fiji Version, NIH, Bethesda, MD, USA) to visualize the fluorescence uptake by cells.

### 2.10. Cellular Release 

The epirubicin intracellular release was investigated by using HPLC analysis. CCRF-CEM cells were grown in 75 cm^2^ culture flasks with RPMI medium (containing 10% FBS and 1% penicillin-streptomycin) to achieve ∼70–80% confluence (1 × 10^7^ cells/mL). At the beginning, the cells were seeded into 6-well plates (1 × 10^7^ in 2 mL of medium per well). The epirubicin-loaded [(WR)_5_C]–GdNPs (5:50 μM, 1:10 ratio) was added to cells in medium. The cells were incubated for 2–72 h at 37 °C. After the incubation time, the cells were collected and centrifuged. The supernatant was removed. The cell pellets were washed by using ice-cold PBS (2 × 5 mL). The cell pellets were extracted using a cocktail containing an equal volume of methanol, chloroform and isopropanol mixture (4:3:1, *v*/*v*/*v*) and filtered through 0.2 μm filters. The solvents were evaporated under N_2_ gas. The sample was dissolved in water and acetonitrile and run through HPLC system connected to UV/Vis detector (490 nm) to measure the released epirubicin. The HPLC protocol was carried out was similar to our previously reported method [29].

### 2.11. Statistical Analysis

To analyze the statistical significance of the dataset, different comparative studies such as cellular uptake study, mechanism of cellular uptake and cell viability assay were performed. Cyclic peptide [(WR)_5_C] and [(WR)_5_C]-GdNPs were compared in different treatment and different datasets were collected. The datasets were used to test ANOVA single factor in MS Excel 2016 to find the *p*-values of comparative datasets. *p*-value below 0.05 was considered as significant for all analyses. If a *p*-value is less than 0.05, it is denoted with one asterisk. If a *p*-value is less than 0.01, it is denoted with two asterisks. If a *p*-value is less than 0.001, it is denoted with three asterisks.

## 3. Results and Discussion

### 3.1. Synthesis of Cell-Penetrating Ligand

**Figure 1:**
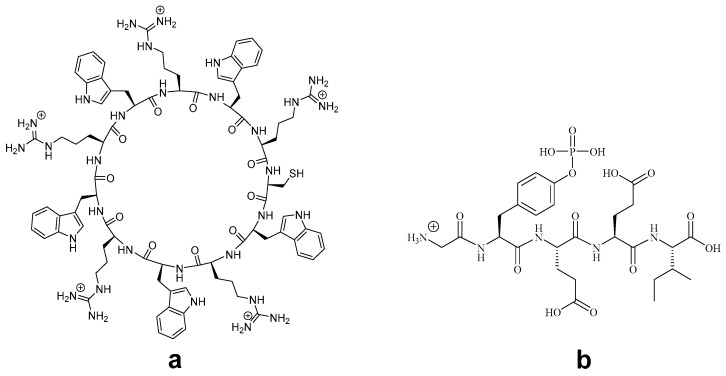
Chemical structures of cyclic peptide [(WR)_5_C] (**a**) and phosphopeptide GpYEEI (**b**).

The cyclic cell-penetrating peptide [(WR)_5_] was selected with the inclusion of a cysteine residue to interact with the metal ion to form nanoparticles. The homochiral cyclic cell-penetrating peptide **a** [(WR)_5_C] was synthesized using solid-phase peptide chemistry according to the previously reported procedure (Figure 1) [30]. The linear side chain protected peptide was assembled using chlorotrityl resin, which was selectively cleaved from solid support to perform N to C terminal cyclization under dilute condition. The cleavage of side chain protecting groups under the acidic condition with scavengers afforded white water-soluble powder showing a mass of 1814.7237 Dalton in MALDI-TOF, corresponding to [M + H]^+^. The peptide was purified by RP-HPLC and lyophilized to obtained solid white powder.

Furthermore, a phosphopeptide was selected as a model cargo molecule to evaluate the efficiency of [(WR)_5_C]-GdNPs as transporters. Phosphopeptides are important probes and used in signaling pathway investigations. However, their intracellular uptake has been limited due to the presence of negatively charged phosphate groups in their structures. Here, GpYEEI was synthesized and used for binding evaluation. GpYEEI is used to study the SH2 domain interaction in the Src tyrosine kinase [31]. To demonstrate the delivery of negatively charged peptide, we selected GpYEEI. The selected cell impermeable phosphopeptide GpYEEI **b** was generated according to the previously reported protocol [31]. The internalization of GpYEEI into cells is a challenging task. Therefore, a sample phosphopeptide GpYEEI was used as a molecular cargo molecule. Further information about the purity could be found in Supplementary Materials. 

### 3.2. Assessment of [(WR)_5_C] for the Formation of Peptide-GdNPs and Their Characterization

An aqueous 10-mM solution of peptide [(WR)_5_C] and GdCl_3_ was prepared and physically mixed at room temperature. There was no change in color of solution after 8–10 h of incubation at room temperature. This resulted in the formation of Peptide–GdNPs. Cyclic peptide containing arginine and tryptophan functioned as both reducing and capping agents for metal nanoparticles. Previously, this combination has been able to generate gold and selenium nanoparticles successfully. This one-pot reaction does not require any additional surface modifying reagents. Mechanistically, tryptophan was found to be a relatively strong reducing agent. Besides, the presence of positive charge in the structure of arginine was found to be a facilitator for this reaction, probably through electrostatic interactions. The presence of one cysteine amino acid was helpful in stabilizing the nanoparticles and facilitating the formation of gadolinium nanoparticles [27].

### 3.3. Transition Electron Microscopy (TEM)

**Figure 2:**
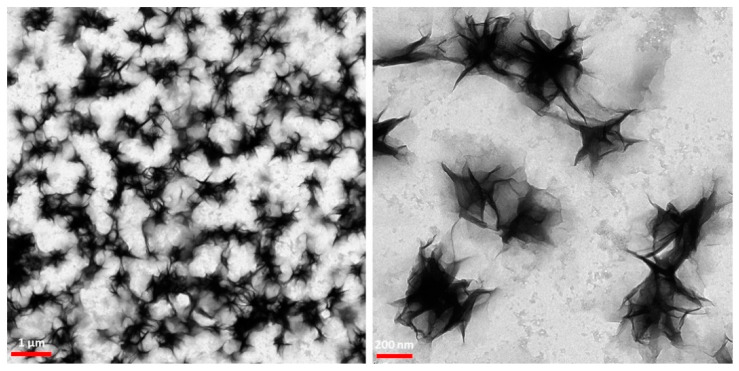
TEM images of [(WR)_5_C]–GdNPs.

To find the size and morphology of [(WR)_5_C]-GdNPs nanoparticles, TEM were performed. The TEM images showed that [(WR)_5_C]-GdNPs formed star-like nanosized structures with an approximate size of 240–260 nm after one-day incubation (Figure 2). Further imaging showed that these star-like particles formed a random network, suggesting that peptides are responsible for holding them together. Similar structures have been reported as coacervates in the literature. Classically, coacervates form through electrostatic attractions between two oppositely charged systems. The structures were found to favor the entropy to become stable. Furthermore, the interactions between positive charge and π-system were reported to form non-classic coacervates [32].

The Gd nanoparticles were surrounded and covered with a layer peptide suggesting the presence of non-covalent or covalent interaction between gadolinium nanoparticles and peptides as a binding ligand. It was previously found that tryptophan amino acid creates a hydrophobic environment that could serve as an effective force to form specific structures. It is worth mentioning that the orientation of amino acids in the structure of the peptide was found to be a significant element in the formation of the morphology and size of these particles. However, more research is required to optimize the size and morphology of these nanoparticles.

### 3.4. Zeta Potential Measurement

To evaluate the binding affinity between peptide-capped nanoparticles and negatively charged phosphopeptides, the surface charge of [(WR)_5_C]-GdNPs was measured before and after loading with the phosphopeptide, GpYEEI. The ratio and concentration were selected to be similar to that of biological assays. Thus, the surface charge of [(WR)_5_C]-GdNPs (50 µM) alone, GpYEEI (5 µM) alone and [(WR)_5_C]-GdNPs loaded GpYEEI mixture (50 µM:5 µM) were obtained. The zeta potential results show that the surface charge of [(WR)_5_C]-GdNPs alone, GpYEEI alone, and [(WR)_5_C]-GdNPs loaded GpYEEI mixture were found to be 24, −2 and 21 mV. These data show that the positive charge of peptide-gadolinium nanoparticles reduced in the presence of the negatively-charged phosphopeptide, suggesting electrostatic interaction is one of the contributing forces in the binding process. All results meet the quality criteria of the Zetasizer instrument.

### 3.5. Encapsulation of Camptothecin by Peptide-GdNPs

**Figure 3:**
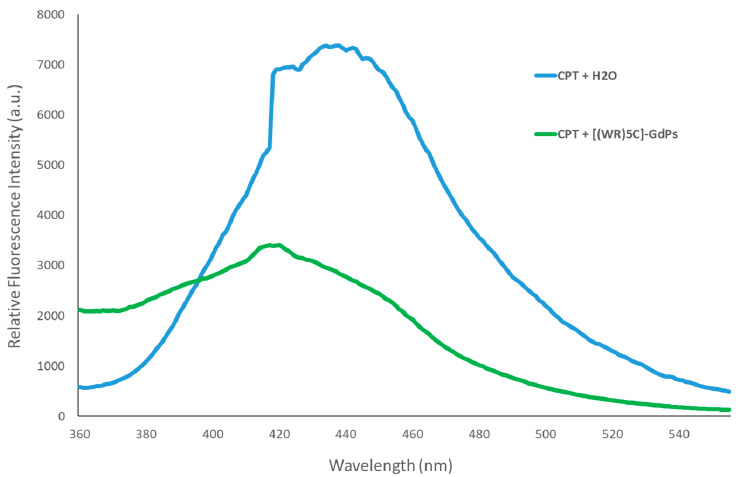
Fluorescence spectra of CPT in the presence of [(WR)_5_C]-GdNPs (1:1 molar ratio) after 2-h incubation.

To validate [(WR)_5_C]-GdNPs for entrapping clinically available drugs, camptothecin (CPT) was chosen as a model drug in fluorescence spectroscopy investigations. CPT is known as a topoisomerase I Inhibitor with a hydrophobic chemical structure [33]. The spectroscopy results show a significant blue shift in maxima’s emission of CPT fluorescence spectra. Due to the interaction of CPT with [(WR)_5_C]-GdNPs, the characteristic CPT’s maxima peak at 432 nm was shifted to 417 nm (Figure 3). The involved hydrophobic forces between CPT and [(WR)_5_C]-GdNPs could possibly be responsible for the observed blue shift of CPT’s peak in the presence of [(WR)_5_C]-GdNPs. In addition, the peak intensity of CPT was reduced significantly due to the self-quenching of bounded drug and also the outcome of the partitioning and entrapment in the hydrophobic pocket generated by the [(WR)_5_C]-GdNPs [19]. The encapsulation findings show that the [(WR)_5_C]-GdNPs were capable of entrapping CPT because of the hydrophobic pockets possibly generated by involved tryptophans.

### 3.6. Cytotoxicity of Peptide-GdNPs

**Figure 4:**
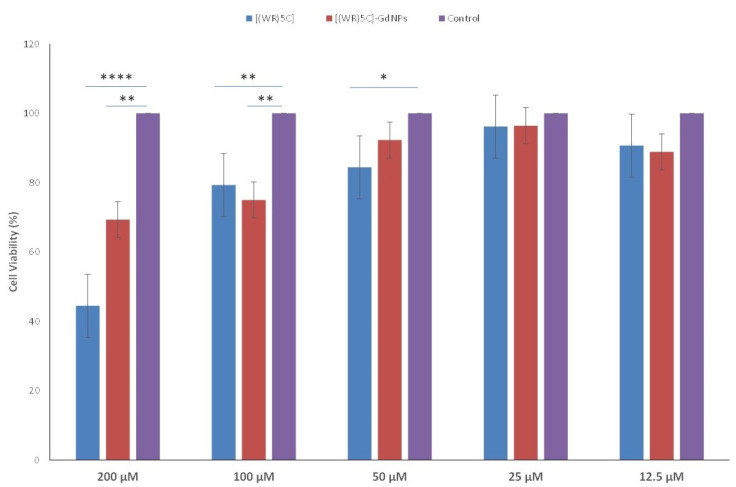
Cytotoxicity of [(WR)_5_C] and [(WR)_5_C]-GdNPs after 24-h incubation. (if *p* < 0.05 then *, if *p* < 0.01 then **, if *p* < 0.0001 then ****).

The cytotoxicity of freshly synthesized [(WR)_5_C]-GdNPs was tested in human leukemia (CCRF-CEM) cells. The concentrations 12.5, 25, 50, 100, and 200 µM were selected. The peptide-capped gadolinium nanoparticles did not show significant toxicity (~93% cell viability at 50 µM) after 24 h of incubation (Figure 4). Therefore, for further cell-based investigations, the concentration of 50 μM was chosen.

### 3.7. Evaluation of Peptide-GdNPs as Molecular Transporters

**Figure 5:**
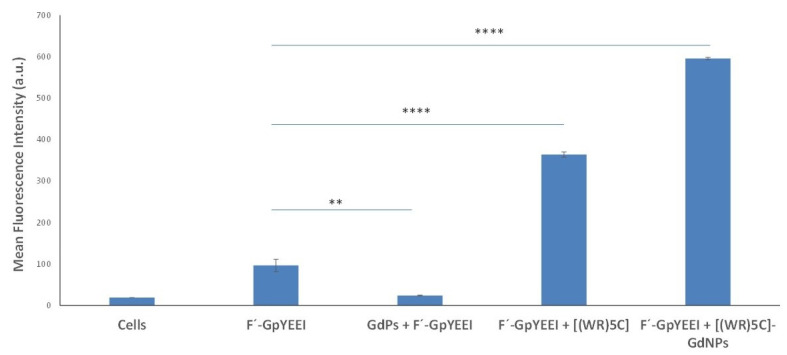
Cellular uptake of F′-GpYEEI in the presence of [(WR)_5_C] and [(WR)_5_C]-GdNPs (50 µM) after 2-h incubation in CCRF-CEM cells. (if *p* < 0.01 then **, if *p* < 0.0001 then ****).

To obtain a sufficient understanding of the potential of [(WR)_5_C]-GdNPs for carrying cargos intracellularly, the flow cytometry method was used. A fluorescence-labeled cell impermeable negatively charged phosphopeptide, F′-GpYEEI, was selected as a cargo model. GpYEEI mimics the pTyr1246 of ErbB2 that binds to the Chk SH2 domain [34]. Traditionally, the delivery of phosphopeptides is a challenging task due to the presence of negatively charged phosphate groups. At the same time, their size is another barrier to enter cells. CCRF-CEM cells were selected for this assay. FACS analysis revealed that the cellular uptake of F′-GpYEEI (5 μM) was enhanced by six folds in the presence of [(WR)_5_C]-GdNPs (50 μM) compared to that of phosphopeptide alone (Figure 5). These data suggest that [(WR)_5_C]-GdNPs may potentially function as a delivery tool to carry F′-GpYEEI intracellularly. The cellular uptake of F′-GpYEEI was not improved in the presence of gadolinium salt solution alone, suggesting that the presence of peptides in the structure of the complex is critical for improving the uptake.

The intracellular uptake of F′-GpYEEI (5 μM) was also improved by peptide alone compared to that of F′-GpYEEI alone. However, the presence of gadolinium enhanced the uptake even further by approximately 1.7 folds in comparison with that of peptide alone. Another control experiment was performed by using the phosphopeptide (F′-GpYEEI) alone. These results show that, although the presence of the peptide is necessary for enhanced delivery efficiency of the carrier, the gadolinium nanoparticles improve the cellular uptake of the drug, suggesting that the uptake of the drug was significantly enhanced, presumably due to the new orientation of amino acids and gadolinium nanoparticles.

**Figure 6:**
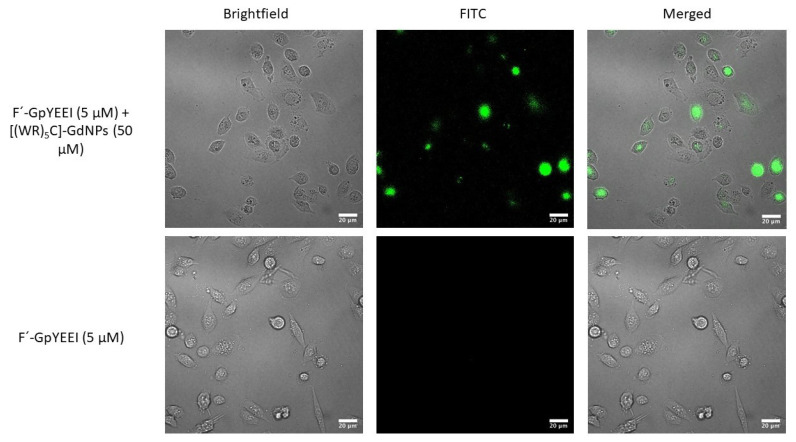
Confocal microscope images of F′-GpYEEI uptake by MCF-7 cells in the presence of [(WR)_5_C]-GdNPs after 2-h incubation.

Confocal microscopy was used to visualize the comparative enhancement of intracellular uptake of F′-GpYEEI (5 μM) in MCF-7 cells. The cells that were treated with only F′-GpYEEI did not exhibit any detectable fluorescence intensity during imaging. The microscopy imaging exhibited higher fluorescence intensity in the presence of F′-GpYEEI [(WR)_5_C]-GdNPs (50 μM) compared to that of phosphopeptide (F′-GpYEEI) alone (Figure 6).

### 3.8. Mechanistic Studies of Cellular Internalization

**Figure 7:**
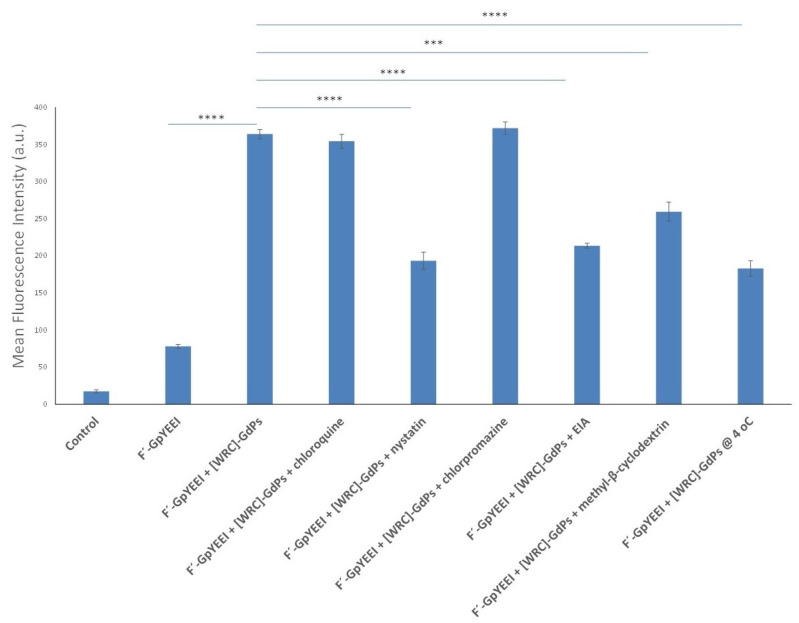
Cellular uptake of F′-GpYEEI loaded [(WR)_5_C]-GdNPs in the absence or presence of different endocytic inhibitors in MCF-7 cells after 2 h. (if *p* < 0.001 then ***, if *p* < 0.0001 then ****).

The mechanism of cellular internalization has always been an area of interest for researchers. When cargos approach the cell membrane, they can enter cells through various mechanisms, including phagocytosis, micropinocytosis and receptor-mediated endocytosis. Receptor-mediated endocytosis (RME) route could include various pathways such as clathrin-mediated, caveolae-mediated and caveolae/clathrin-independent endocytosis [33,34,35]. To find the mechanism of uptake of cargo-loaded-peptide-capped gadolinium nanoparticles, quantitative FACS studies were used. The fluorescence intensity of F′-GpYEEI loaded [(WR)_5_C]-GdNPs (5:50 μM) was tested quantitatively in the presence of several endocytic inhibitors, namely chloroquine, nystatin, chlorpromazine, 5-(*N*-ethyl-*N*-isopropyl)-amiloride (EIA) and methyl-β-cyclodextrin, by using FACS (Figure 7).

As showed in Figure 7, no significant decrease of the fluorescence signal of F′-GpYEEI loaded [(WR)_5_C]-GdNPs was observed in MCF-7 cells when chloroquine, chlorpromazine and methyl-β-cyclodextrin were present after 2 h of incubation at 37 °C. These findings reveal that the clathrin-mediated or caveolae-mediated endocytosis and phagocytosis are not majorly responsible pathways for the uptake. Macropinocytosis was found to be an important pathway because the uptake of the complex was reduced in the presence of nystatin and EIA by 40% and 45%, respectively. The FACS results in Figure 7 reveal that, although these two inhibitors reduced the internalization of the cargo significantly, they could not shut down the pathway completely, suggesting other pathways could also be involved here in transporting F′-GpYEEI loaded [(WR)_5_C]-GdNPs intracellularly. Additionally, the cellular uptake of F′-GpYEEI loaded [(WR)_5_C]-GdNPs was reduced by 50% when cells were incubated at 4 °C. These results potentially suggest that the mechanism of uptake is a partially energy-dependent pathway.

Gadolinium nanoparticles have already been used for MRI imaging; thus, they are promising platforms for delivery applications. However, the functionalization of the surface of gadolinium nanoparticles with [(WR)_5_C] peptide make them more intractable with the cell membrane. The nature of this interaction consists of electrostatic interactions and hydrophobic forces generated by arginine and tryptophan amino acids, respectively. The functional groups in the structure of these amino acids could induce binding affinity with the negatively charged phospholipids in the cell membrane. This interaction follows by hydrophobic forces induced via indole groups available in tryptophan amino acid leading to higher intracellular penetration due to disturbed lipid bilayer in the structure of the cell membrane. The last step of this process is peptide internalization into the membrane to enhance the cellular uptake of the cargo. The mechanism of cellular internalization of the peptide was found to be partially energy independent. When the peptide was mixed with GdNPs, the mechanism of uptake favors the energy-dependent mechanism, suggesting gadolinium nanoparticles are responsible for the uptake. Thus, the gadolinium nanoparticles influence the mechanism of uptake. However, more investigations are needed to find the detailed mechanism of cell entry of [(WR)_5_C]-GdNPs.

### 3.9. Intracellular Release of Epirubicin

To investigate the intracellular drug release in a timely manner, epirubicin was selected to be a model drug. Analytical HPLC technique was used to study the release of epirubicin intracellularly by [(WR)_5_C]-GdNPs-epirubicin complex in CCRF-CEM cells. CCRF-CEM cells (1.2 × 10^7^) were treated by epirubicin (5 μM)-loaded [(WR)_5_C]-GdNPs (50 μM) for different incubation times (12–48 h). The amount of epirubicin was measured using HPLC at 490 nm. HPLC data show that [(WR)_5_C]-GdNPs-epirubicin releases epirubicin in a time-dependent manner: approximately 15% and 60% of epirubicin was released intracellularly within 12 and 48 h, respectively. These data suggest the sustained release of epirubicin contributes to the overall activity of the epirubicin-loaded [(WR)_5_C]-GdNPs as a potential prodrug.

### 3.10. Time-Dependent Antiproliferative Activity Assay

**Figure 8:**
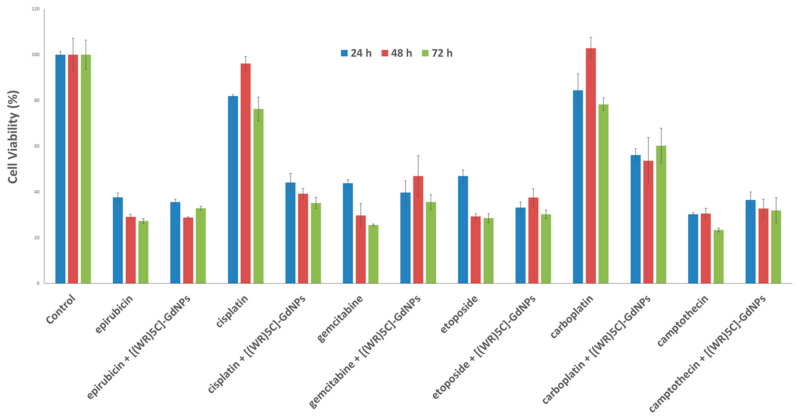
Antiproliferative activity of epirubicin, cisplatin, gemcitabine, etoposide, carboplatin and camptothecin (5 µM) in CCRF-CEM cells in the absence and presence of [(WR)_5_C]-GdNPs. The experiments were conducted in triplicate (*n* = 3).

Several anticancer drugs such as epirubicin, cisplatin, gemcitabine, etoposide, carboplatin and camptothecin were evaluated for their antiproliferative activities at different time periods, including 24, 48 and 72 h. The results of antiproliferative assay exhibit that the potency of epirubicin, cisplatin, gemcitabine, etoposide, carboplatin and camptothecin (5 µM) was enhanced by 6%, 41%, 10%, 2%, 18% and 8%, respectively, in the presence of [(WR)_5_C]-GdNPs (50 μM) in CCRF-CEM cells after 72 h of incubation (Figure 8). The improved antiproliferative activity of cisplatin and carboplatin could be due to the chelating of the thiol group of cysteine with these anticancer agents or binding of metals to peptides as GdNPs. As evident in Figure 8, the antiproliferative activity of these anticancer drugs-loaded [(WR)_5_C]-GdNPs was generally time-dependent in most cases. This effect could happen for various reasons. This could presumably occur through higher cellular uptake of drugs. Additionally, although gadolinium nanoparticles are bio-friendly, the accumulation of them in target tissue can potentially induce toxicity. Here, the peptide functions as a ligand to neutralize the toxicity of gadolinium nanoparticles upon the internalization into cells. However, when the complex starts being metabolized inside cells, the gadolinium nanoparticles assist the anticancer drugs through synergistic effect, leading to a higher antiproliferative activity compared to that of drug alone.

## 4. Conclusions

A new metal-containing drug delivery system was designed using gadolinium nanoparticles and cyclic peptides containing tryptophan, cysteine and arginine amino acids. [(WR)_5_C]-GdNPs exhibited notable interaction with CPT through non-covalent interactions. The complex functioned as a carrier to deliver a negatively charged cell-impermeable phosphopeptide (F′-GpYEEI) into cells. The uptake efficiency of the molecular cargo in the presence of the carrier complex was confirmed by confocal microscopy. Time-dependent antiproliferative studies showed that multiple anticancer drugs such as epirubicin, cisplatin, gemcitabine, etoposide, carboplatin and camptothecin can inhibit the proliferation of CCRF-CEM cells after 72 h of incubation by 6%, 41%, 10%, 2%, 18% and 8%, respectively, when combined with [(WR)_5_C]-GdNPs (50 μM). This investigation provides further understanding of how to take advantage of gadolinium for the efficient delivery of cell-impermeable cargo molecules in combination with peptides. The peptide reduces the toxicity of the metal, while also enhancing the intracellular uptake.

## Supplementary Materials

The following are available online at https://www.mdpi.com/1999-4923/12/9/792/s1, the MALDI and ESI mass spectra and HPLC chromatograms are provided.
