# Supplementary material for: Cyclic Peptide-Gadolinium Nanoparticles for Enhanced Intracellular Delivery
Source: Pharmaceutics. 2020 Aug 21;12(9):792. doi: 10.3390/pharmaceutics12090792 (PMC7557599; doi:10.3390/pharmaceutics12090792)
Supplement: Supplementary file 1 [file pharmaceutics-12-00792-s001.pdf]

pharmaceutics

Supplementary Materials: Cyclic Peptide-Gadolinium Nanoparticles for Enhanced Intracellular Delivery

Amir Nasrolahi Shirazi [1,*], Shang Eun Park [2], Shirin Rad [1], Luiza Baloyan [1], Dindyal Mandal [3], Muhammad Imran Sajid [2,4], Ryley Hall [2], Sandeep Lohan [2], Khalid Zoghebi [2], Keykavous Parang [2] and Rakesh Kumar Tiwari [2,*]

[1] Department of Pharmaceutical Sciences, College of Pharmacy, Marshall B. Ketchum University, Fullerton, CA 92831, USA; ShirinRad.PH23@ketchum.edu (S.R.); LuizaBaloyan.PH23@ketchum.edu (L.B.)
[2] Center for Targeted Drug Delivery, Department of Biomedical and Pharmaceutical Sciences, Chapman University School of Pharmacy, Harry and Diane Rinker Health Science Campus, Irvine, CA 92618, USA; park327@mail.chapman.edu (S.E.P.); sajid@chapman.edu (M.I.S.); hall222@mail.chapman.edu (R.H.); lohan@chapman.edu (S.L.); zoghe101@mail.chapman.edu (K.Z.); parang@chapman.edu (K.P.)
[3] School of Biotechnology, KIIT Deemed to be University, Bhubaneswar 751024, India; dmandal@kiitbiotech.ac.in
[4] Faculty of Pharmacy, University of Central Punjab, Lahore 54000, Pakistan
* Correspondence: ashirazi@ketchum.edu (A.N.S.); tiwari@chapman.edu (R.K.T.); Tel.: +1-714-449-7497 (A.N.S.); +1-714-516-5483 (R.K.T.); Fax: +1-714-872-5706 (A.N.S); +1-714-516-5481 (R.K.T.);

**Table of Contents**	**Page**
1. Analytical HPLC chromatogram of [(WR)$_5$C]	S3
2. MALDI-TOF mass spectra of [(WR)$_5$C]	S4
3. Analytical HPLC chromatogram of GpYEEI	S5
4. ESI-TOF mass spectra of GpYEEI	S6

1. Analytical HPLC chromatogram of [(WR)$_5$C]

The analytical HPLC was performed on a Hitachi analytical HPLC system using a C18 Shimadzu Premier 3 µm column (150 cm × 4.6 mm) using a gradient solvent system, and a flow rate of 1 mL/min with detection at 214 nm.

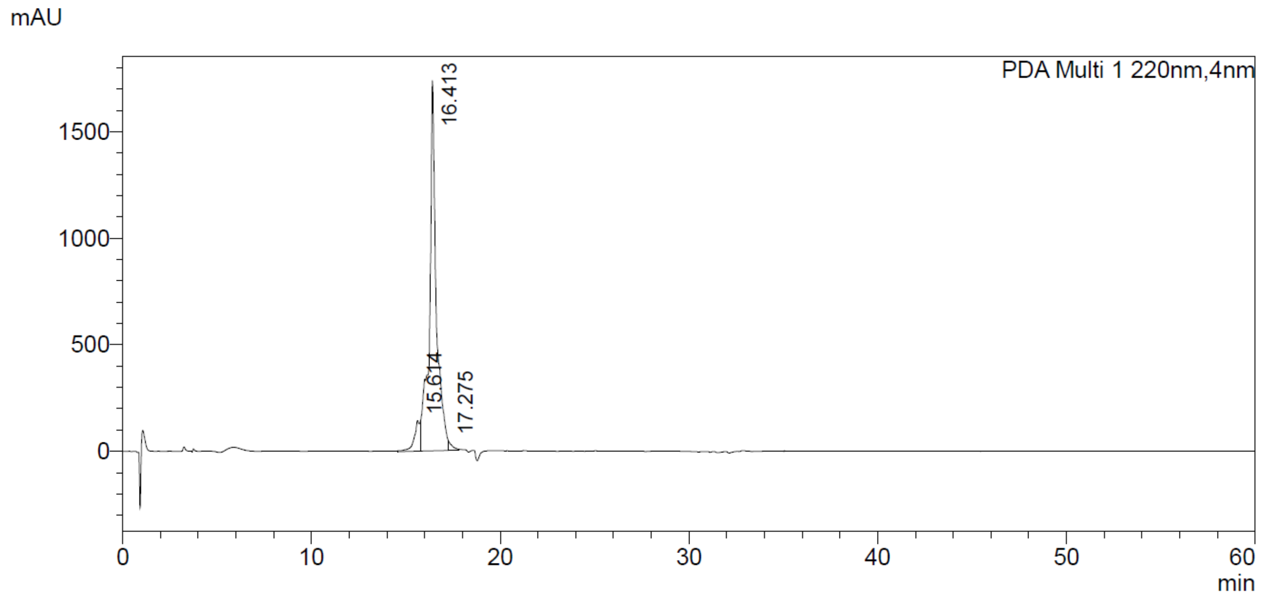

Figure 1. The analytical HPLC chromatogram of [(WR)$_5$C].

2. MALDI-TOF mass spectra of [(WR)$_5$C]

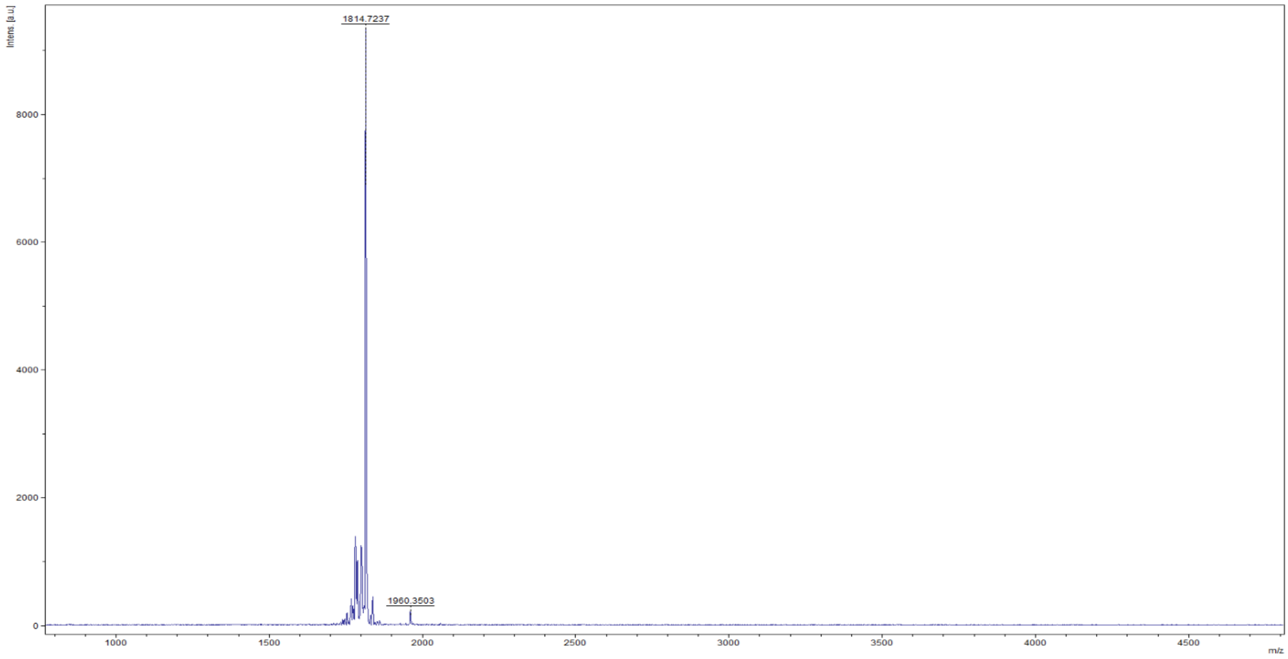

Figure 2. The mass spectra of [(WR)$_5$C] using the MALDI-TOF system.

3. Analytical HPLC chromatogram of GpYEEI

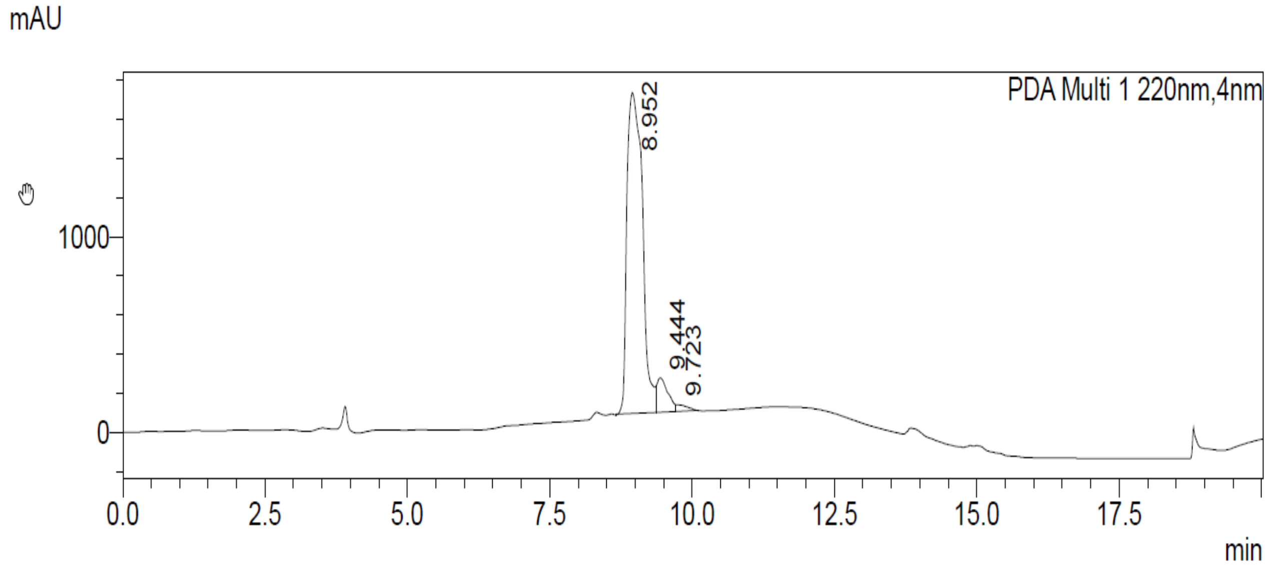

Figure 3. The HPLC chromatogram of GpYEEI.

4. ESI-TOF mass spectra of GpYEEI

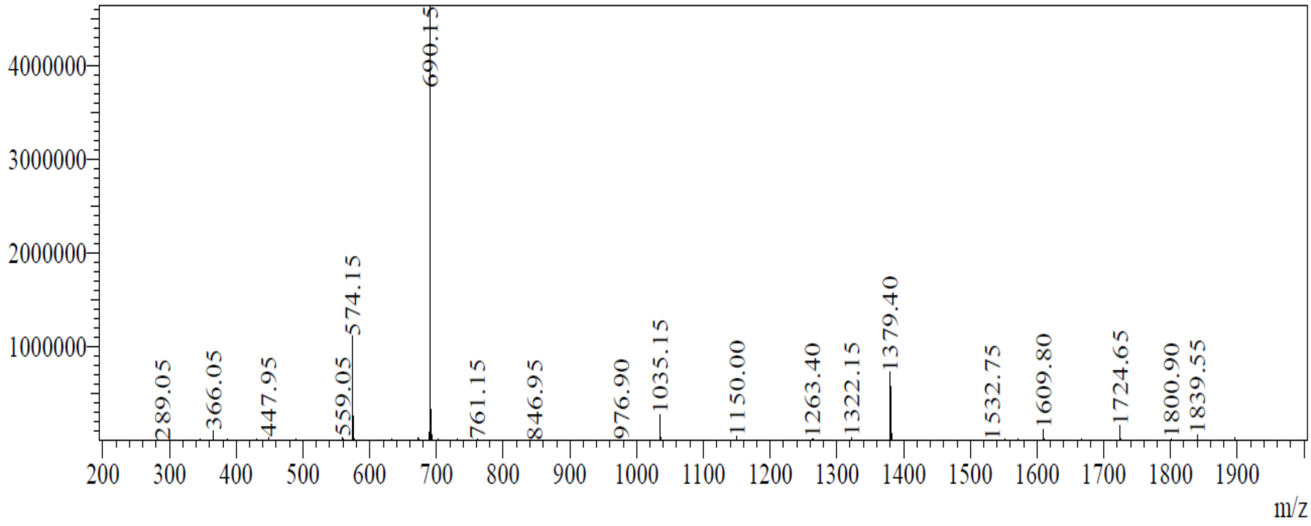

Figure 4. The mass spectra of GpYEEI using qTOF ESI system.
